# Fatal Hyperkalemia Following Reduction of a Strangulated Obturator Hernia: A Pitfall in Assessing Bowel Viability

**DOI:** 10.70352/scrj.cr.26-0515

**Published:** 2026-08-28

**Authors:** Takanori Kinashi, Toshihiro Tsuruda

**Affiliations:** 1 Department of Surgery, Nanbu Hospital, Miyazaki, Miyazaki, Japan; 2 Department of Hemo-Vascular Advanced Medicine, Faculty of Medicine, University of Miyazaki, Miyazaki, Miyazaki, Japan

**Keywords:** obturator hernia, reperfusion injury, hyperkalemia, small bowel obstruction, case report

## Abstract

**INTRODUCTION:**

Obturator hernia is a rare cause of small-bowel obstruction and is frequently associated with strangulation. Even after successful reduction, bowel ischemia–reperfusion injury may occur and result in life-threatening metabolic complications.

**CASE PRESENTATION:**

An 87-year-old woman presented with a 3-week history of nausea and vomiting. CT revealed a right obturator hernia with incarceration of a small-bowel loop. US-guided manual reduction was successfully performed. Contrast-enhanced CT obtained immediately after reduction demonstrated preserved bowel-wall enhancement with circumferential mural thickening. The serum potassium level at presentation was 4.6 mmol/L. Despite apparently preserved bowel perfusion on CT, she continued to experience abdominal pain, tachypnea, and progressively deteriorating consciousness. Approximately 5 h after reduction, she developed sudden bradycardia followed by cardiac arrest. Continuous electrocardiographic monitoring showed progression from sinus rhythm to loss of P waves, QRS widening, and finally a sine-wave pattern. Laboratory testing during resuscitation revealed severe hyperkalemia (8.5 mmol/L). Despite intensive resuscitative efforts, the patient died.

**CONCLUSIONS:**

Preserved bowel-wall enhancement after reduction of a strangulated obturator hernia does not necessarily indicate bowel viability. A prolonged clinical course, persistent symptoms or systemic abnormalities, and residual CT abnormalities such as bowel-wall thickening and ascites should be considered together when assessing bowel viability after reduction. Such findings should prompt careful reassessment, close monitoring, and consideration of early surgical exploration despite preserved bowel-wall enhancement.

## Abbreviations

AST
: aspartate aminotransferase
CPR
: cardiopulmonary resuscitation
FROGS
: four-hand reduction for obturator hernia with the guidance of sonography
NG
: nasogastric

## INTRODUCTION

Obturator hernia is an uncommon cause of small-bowel obstruction and occurs most frequently in older, thin, multiparous women.^1,2)^ Because the obturator canal is narrow, incarceration and strangulation are common, and delayed diagnosis may result in bowel ischemia and necrosis. US-guided manual reduction has increasingly been adopted as an initial treatment strategy in carefully selected patients with incarcerated obturator hernia.^3–5)^ However, despite successful reduction, the reduced bowel may still have sustained substantial ischemic injury. Reperfusion of severely ischemic bowel may trigger metabolic derangements, including severe hyperkalemia, potentially resulting in fatal arrhythmias.^6)^ We report a case of fatal hyperkalemia most likely triggered by bowel ischemia–reperfusion injury after successful manual reduction of a strangulated obturator hernia and discuss the challenges in assessing bowel viability after reduction.

## CASE PRESENTATION

**Fig. 1:**
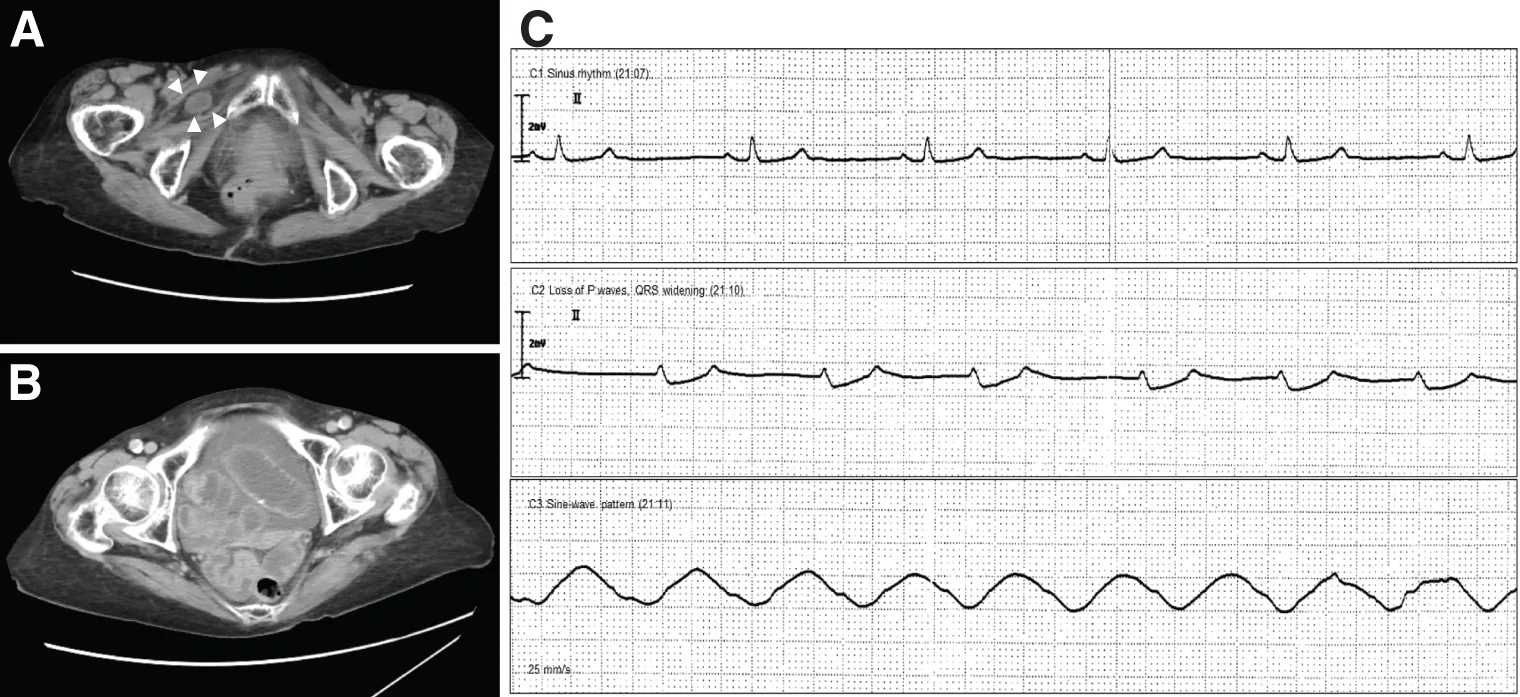
(**A**) Non-contrast CT showing a small-bowel loop incarcerated within the right obturator canal. (**B**) Contrast-enhanced CT obtained immediately after reduction, showing preserved bowel-wall enhancement, mildly increased ascites, and circumferential mural thickening in the portal venous phase. (**C**) Continuous electrocardiographic monitoring demonstrating progression from sinus rhythm to loss of P waves and widening of the QRS complexes, followed by a characteristic sine-wave pattern consistent with severe hyperkalemia.

An 87-year-old woman (height, 138 cm; weight, 38 kg; BMI, 20.0 kg/m^2^) presented with a 3-week history of nausea and vomiting. During the preceding 10 days, she developed poor oral intake, progressive lower-extremity edema, and a 4-kg weight loss (from 42 to 38 kg). Her medical history included hypertension and chronic constipation. Her regular medications included amlodipine (5 mg/day), spironolactone (25 mg/day), furosemide (20 mg/day), and sodium picosulfate (5 mg/day). She had no history of previous abdominal surgery. On physical examination, her blood pressure was 137/77 mmHg, heart rate was 88 beats/min, body temperature was 37.0°C, oxygen saturation was 98% on room air, and abdominal distension was noted. Intravenous fluid administration with Lactec injection (Otsuka Pharmaceutical Factory, Tokushima, Japan) was initiated at 250 mL/h to correct dehydration. Non-contrast CT was initially performed because of the patient’s advanced age and mildly impaired renal function (**Table 1**). Non-contrast CT demonstrated a right obturator hernia with incarceration of a small-bowel loop between the pectineus and obturator muscles without evidence of free intraperitoneal air (**Fig. 1A**). US-guided manual reduction was successfully performed.

**Table 1:** Comparison of laboratory findings

Laboratory parameter	At presentation	During resuscitation	Reference range
Time	15:07	21:29	
WBC (/μL)	8420	7000	3500–9700
Hemoglobin (g/dL)	14.7	13.2	11.2–15.2
Platelets (×10^4^/μL)	34.9	16.2	14.0–37.9
BUN (mg/dL)	52.9	43.0	8.0–20.0
Creatinine (mg/dL)	0.85	1.3	0.46–0.82
Sodium (mmol/L)	136	137	135–145
Potassium (mmol/L)	4.6	8.5	3.5–5.0
Chloride (mmol/L)	93	106	98–108
CK (U/L)	77	96	50–210
AST (U/L)	37	116	10–40
ALT (U/L)	26	84	5–45
Amylase (U/L)	61	611	39–134
CRP (mg/dL)	0.54	0.60	0.00–0.30
Glucose (mg/dL)	91	465	70–109

Values at presentation were obtained before manual reduction. Values during resuscitation were obtained from an arterial blood sample.

ALT, alanine aminotransferase; AST, aspartate aminotransferase; BUN, blood urea nitrogen; CK, creatine kinase; CRP, C-reactive protein; WBC, white blood cell

**Fig. 2:**
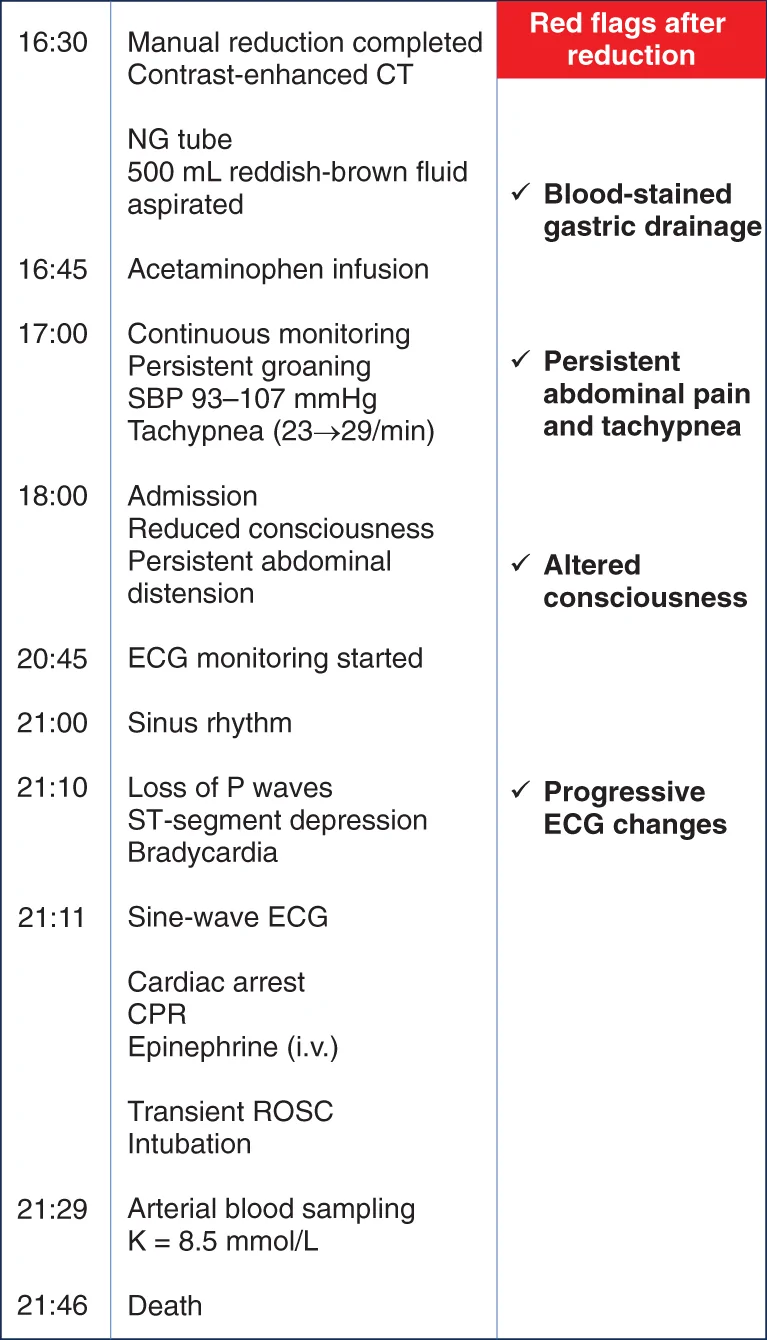
Clinical timeline after successful manual reduction of an incarcerated obturator hernia illustrating the sequence of CT assessment, persistent clinical warning signs despite apparently preserved bowel-wall enhancement, electrocardiographic progression, CPR, and confirmation of severe hyperkalemia. CPR, cardiopulmonary resuscitation; ECG, electrocardiogram; i.v., intravenous; NG, nasogastric; ROSC, return of spontaneous circulation; SBP, systolic blood pressure

Immediately after reduction, contrast-enhanced CT demonstrated mildly increased ascites, preserved bowel-wall enhancement, and circumferential mural thickening, without free intraperitoneal air or high-attenuation ascites suggestive of hemorrhagic fluid (**Fig. 1B**). Based on these findings, urgent laparoscopic hernia repair was planned for the following morning. The patient was admitted for close observation because delayed bowel ischemia or perforation may occur after manual reduction. **Figure 2** illustrates a timeline summarizing the post-reduction clinical course. Blood-stained NG drainage was noted, and the patient continued to experience abdominal pain, groaning, tachypnea, and progressive deterioration of consciousness. Approximately 5 h after successful reduction, the patient developed bradycardia and became unresponsive. CPR was initiated. Despite resuscitative efforts, the patient died. Continuous electrocardiographic monitoring showed the rapid progression from sinus rhythm to loss of P waves and widening of the QRS complexes, followed by a sine-wave pattern (**Fig. 1C**). An arterial blood sample obtained during resuscitation revealed severe hyperkalemia (8.5 mmol/L).

## DISCUSSION

Obturator hernia is associated with a high risk of bowel strangulation because of the narrow hernia orifice. Recent weight loss may have contributed to atrophy of the protective preperitoneal fat within the obturator canal, thereby predisposing the patient to obturator hernia. At our institution (Nanbu Hospital), manual reduction using the FROGS method is indicated only in patients without signs of shock or bowel necrosis on CT or ultrasonography.^5,7,8)^ Following successful reduction, patients are admitted for close observation and undergo urgent laparoscopic repair after reassessment of bowel viability. Although the amount of ascites increased mildly after reduction, the fluid showed no CT features suggestive of hemoperitoneum, and no free intraperitoneal air or microbubbles were identified. These post-reduction CT findings were reviewed in real time with a radiologist and, together with the preserved bowel-wall enhancement, were interpreted as showing no definitive evidence of transmural bowel necrosis or perforation at that time. However, this case demonstrates that such imaging findings should always be interpreted together with persistent clinical abnormalities. In retrospect, these persistent clinical abnormalities were initially attributed to the residual effects of prolonged bowel obstruction but should instead have been recognized as important warning signs (**Fig. 2**). Our findings are consistent with those of Huszty et al., who reported that CT findings suggestive of bowel ischemia, particularly bowel-wall thickening and ascites, were associated with the need for bowel resection.^9)^ Gokon et al. reported that patients requiring bowel resection had a significantly longer duration of symptoms than those without resection (88 vs. 36 h), with a symptom duration of ≥72 h associated with bowel non-viability.^3)^ Similarly, Ishiguro et al. identified a symptom duration of ≥36 h as an independent predictor of bowel resection.^10)^ In the present case, nausea and vomiting had persisted for 3 weeks, with markedly reduced oral intake for approximately 10 days. Although the exact duration of bowel incarceration could not be determined from these symptoms, this prolonged clinical course, together with persistent bowel-wall thickening and increased ascites after reduction, should, in retrospect, have raised greater concern for ongoing bowel injury despite preserved bowel-wall enhancement. These observations emphasize that bowel viability after reduction should be assessed by integrating the duration and evolution of symptoms, persistent clinical abnormalities, and CT findings, rather than relying on bowel-wall enhancement alone. If ischemia–reperfusion injury was indeed the underlying mechanism in the present case, reactive hyperemia and increased vascular permeability may have contributed to mucosal damage and may have promoted potassium release into the systemic circulation.^6,11)^ This may ultimately lead to hyperkalemia-related arrhythmias. Severe hyperkalemia is characterized by electrocardiographic changes, including loss of P waves, progressive widening of the QRS complex, and eventual sine-wave morphology, which may precede ventricular fibrillation or asystole.^12,13)^ In the present case, electrocardiographic monitoring showed a typical progression of hyperkalemia-related changes within minutes. Continuous hemodynamic and electrocardiographic monitoring may be warranted in patients with persistent systemic abnormalities after reduction of prolonged bowel incarceration.

We acknowledge that the serum potassium concentration was measured from an arterial blood sample obtained during CPR. Therefore, circulatory collapse and accompanying metabolic derangements may have contributed to the severity of hyperkalemia. However, the patient had a normal serum potassium concentration at presentation (**Table 1**), and alternative causes of abrupt severe hyperkalemia, including advanced renal failure, rhabdomyolysis, and pseudohyperkalemia were not supported by the clinical or laboratory findings (**Table 2**). Additionally, chronic treatment with spironolactone may have increased the patient’s susceptibility to hyperkalemia; however, the abrupt rise following reduction suggests that spironolactone alone was unlikely to explain the clinical course. The close temporal relationship between reduction of the strangulated bowel, the characteristic post-reduction CT findings, and the preceding continuous electrocardiographic progression characteristic of hyperkalemia suggests that bowel ischemia–reperfusion injury was the most plausible initiating mechanism. To place this case in clinical context, we summarized previously published clinically relevant reports (**Table 3**).^6,14)^ Taken together, this case underscores that persistent clinical deterioration after successful reduction should outweigh apparently reassuring CT findings when determining the need for urgent surgical intervention.

**Table 2:** Differential diagnosis of severe hyperkalemia in the present case

Possible cause	Findings in the present case	Interpretation
Acute kidney injury	Cr 0.85 → 1.3 mg/dL	Unlikely as the sole cause
Rhabdomyolysis	CK 77 → 96 U/L	Unlikely
Drug-induced hyperkalemia	Spironolactone (25 mg/day)	Unlikely as the sole cause
Pseudohyperkalemia	No hemolysis reported; no marked thrombocytosis/leukocytosis	Unlikely
Ischemia–reperfusion injury	Temporal relationship, CT findings, ECG progression	Most plausible

The proposed mechanism of ischemia–reperfusion injury is based on the overall clinical and imaging findings rather than pathological confirmation.

CK, creatine kinase; Cr, creatinine; ECG, electrocardiogram

**Table 3:** Published reports relevant to ischemia–reperfusion injury and hyperkalemia associated with incarcerated bowel

Study	Hernia	Release/reduction	Potassium	Arrhythmia	Outcome
Allford et al.,^14)^	Inguinal	Surgical release of incarcerated bowel	Normal	VT/VF	Death
Xu et al.,^6)^	Inguinal	Intraoperative reduction, then resection	6.1 mEq/L at arrest	SVT → asystole	Survived
Present case	Obturator	Manual reduction	8.5 mmol/L	P-wave loss → QRS widening → sine wave →asystole	Death

Only clinically relevant reports describing hyperkalemia or reperfusion-associated arrhythmias after incarcerated bowel were included.

SVT, supraventricular tachycardia; VF, ventricular fibrillation; VT, ventricular tachycardia

### Limitations

This report has several limitations. Histopathological confirmation of ischemia–reperfusion injury was unavailable because neither bowel resection nor autopsy was performed. Furthermore, arterial blood gas analysis, including pH, bicarbonate, base excess, and lactate measurements, was not available during nighttime emergency care at our institution. Consequently, the degree of metabolic acidosis accompanying the severe hyperkalemia could not be evaluated directly. Although direct metabolic assessment was unavailable, elevations in serum amylase and AST were consistent with systemic tissue injury associated with prolonged bowel ischemia and subsequent hemodynamic collapse (**Table 1**). Therefore, the proposed mechanism of bowel ischemia–reperfusion injury should be interpreted as the most plausible explanation based on the clinical course rather than a pathologically confirmed diagnosis.

## CONCLUSIONS

Clinical assessment should take precedence over apparently reassuring contrast-enhanced CT findings when determining bowel viability after reduction of a strangulated obturator hernia. Persistent clinical deterioration should prompt immediate reassessment and consideration of early surgical exploration. Surgeons should recognize bowel ischemia–reperfusion injury as a potentially fatal complication that may result in severe hyperkalemia and fatal arrhythmias.

## DECLARATIONS

### Use of artificial intelligence (AI) tools

No AI tools were used in research development, but it was used to improve the readability of the manuscript.

### Availability of data and materials

Data are available on request from the corresponding author.
